# Genome-wide identification, phylogeny, and expression profiling analysis of shattering genes in rapeseed and mustard plants

**DOI:** 10.1186/s43141-022-00408-2

**Published:** 2022-08-18

**Authors:** Mahideen Afridi, Khurshid Ahmad, Shahana Seher Malik, Nazia Rehman, Muhammad Yasin, Shujaul Mulk Khan, Adil Hussain, Muhammad Ramzan Khan

**Affiliations:** 1 National Centre for Bioinformatics, Quaid-I-Azam University, Islamabad, 45320 Pakistan; 2 Department of Biological Sciences, International Islamic University, Islamabad, 44000 Pakistan; 3 Department of Biology, College of Science, United Arab Emirates University, 15551 Al Ain, United Arab Emirates; 4 National Institute for Genomics and Advanced Biotechnology, National Agricultural Research Center, Park Road, Islamabad, 44000 Pakistan; 5 Department of Plant Sciences, Quaid-I-Azam University, Islamabad, 45320 Pakistan; 6 Food and Biotechnology Research Centre (FBRC), Pakistan Council of Scientific and Industrial Research (PCSIR) Laboratories Complex, Ferozepur Road, Lahore, Punjab 56400 Pakistan

**Keywords:** Rapeseed, Indian mustard, *Brassica* Fruit, Shattering genes, Mads box, Oil seed

## Abstract

**Background:**

Non-synchronized pods shattering in the *Brassicaceae* family bring upon huge yield losses around the world. The shattering process was validated to be controlled by eight genes in *Arabidopsis*, including *SHP1*, *SHP2*, *FUL*, *IND*, *ALC*, *NAC*, *RPL*, and *PG.* We performed genome-wide identification, characterization, and expression analysis of shattering genes in *B.napus* and *B. juncea* to gain understanding into this gene family and to explain their expression patterns in fresh and mature siliques.

**Results:**

A comprehensive genome investigation of *B.napus* and *B.juncea* revealed 32 shattering genes, which were identified and categorized using protein motif structure, exon-intron organization, and phylogeny. The phylogenetic study revealed that these shattering genes contain little duplications, determined with a distinct chromosome number. Motifs of 32 shattering proteins were observed where motifs1 and 2 were found to be more conserved. A single motif was observed for other genes like Br-nS7, Br-nS9, Br-nS10, Br-jS21, Br-jS23, Br-jS24, Br-jS25, and Br-jS26. Synteny analysis was performed that validated a conserved pattern of blocks among these cultivars. RT-PCR based expressions profiles showed higher expression of shattering genes in *B. juncea* as compared to *B.napus*. *SHP1*, *SHP2*, and *FUL* gene were expressed more in mature silique. *ALC* gene was upregulated in fresh silique of *B. napus* but downregulation of *ALC* were observed in fresh silique of *B. juncea*.

**Conclusion:**

This study authenticates the presence of shattering genes in the local cultivars of *Brassica*. It has been validated that the expression of shattering genes were more in *B. juncea* as compared to *B.napus*. The outcomes of this study contribute to the screening of more candidate genes for further investigation.

## Background

*Brassicaceae* is one of the important families, with ~ 360 genera and 3700 species around the globe [1]. Species from this family are very significant from an economic, and agricultural point of view. A few examples of species from this family are *Brassica napus*, *Brassica juncea* (oilseed crops); *Brassica oleracea* (cabbage, cauliflower, kale, broccoli); *Brassica rapa* (turnip, leaf vegetable); *Raphanus sativus* (vegetable); and *Arabidopsis thaliana* (model plant). The most cultivated species of the *Brassica* genus includes those with three diploid genomes like *B. nigra* (BB, 2n =16) *B. oleracea* (CC, 2n = 18), and *B. rapa* (AA, 2n = 20), together with three amphidiploid species like *B. napus* (AACC, 2n = 38) *B. carinata* (BBCC, 2n = 34), and *B. juncea* (AABB, 2n = 36). Hybridization and cytogenetic studies have determined that amphidiploid species are natural hybrids of diploids and all species are interconnected [2].

Non-synchronous pod shattering remained a major problem of *Brassica* that results in yield loss. It also causes seed loss due to the dispersion of a silique following complicated physiological and biological mechanisms [3]. However, premature and unsynchronized pods shattering like the dehiscence results in a huge loss in crop yield [4]. Pod’s shattering occurs when the adhesions among walls change into fragile and internal forces apply them to the moveable position [5]. Seed valves are responsible for the attachment and internal force creation that contributes to the necessary protection of the seed [6]. Seeds of *B. juncea* and *B. napus* are very important equally 14% of oil around the world is produced by these crops. Moreover, rapeseed is considered the third most important oilseed crop worldwide [7]. Distinct nutrients and biological molecules are reported to be involved in the evolution of shattering in canola [8].

Previous investigation over shattering revealed that shattering occurred because of molecular components excess production and enrichment in the valve margin and cellar portion around the pods in siliques. Lignin and cellulose play a key role in the hardening of pod walls, which lowers water content during the later development stages of rapeseed and *Brassica* species [9]. The shattering mechanism of *B. napus* and *B. juncea* are controlled by eight different genes, like *SHATTERPROOF1 (SHP1)*, *SHATTERPROOF2 (SHP2) FRUITFULL (FUL), INDEHISCENT (IND)*, *ALCATRAZ (ALC)*, *NAC*, *(NST1* and *NST2) REPLUMLESS (RPL)*, and *POLYGlACTOURANAZE* [10]. For the development of shattering genes, distinct transcription factor binding sites are involved which are important both structurally and functionally [11]. Other genes like *SHP1/2, FUL, IND*, *ALC*, *NAC*, *RPL*, and *PG* of canola and Indian mustard were also reported [12]. In one study, a comparative analysis was performed to unveil the genomic maintenance for the evolutionary and functional correlation among shattering genes *SHP1/2*, *FUL*, *IND*, *ALC*, *NAC*, *RPL*, and *PG* [13] having functional and genetic conservation among them. The pattern of conservation in these shattering gene sequences was also found with comparative synteny approach by Krzywinski et al. [14].

The most desirable solution to the shattering problem of *B. napus* and *B. juncea* is to delay pod shattering by knocking out *SHPS* genes and stimulating the expression of *FUL* up to the susceptible crop is ready for harvesting. Therefore, before developing genome modified plants it is essential to study these genes elaborately in local plants *B.napus* and *B. juncea*. Therefore, this study was carried out to identify the orthologous of shattering genes in the local cultivars of *B. napus* and *B. juncea* and to study their expression pattern in fresh and mature siliques. This study further identified the syntenic and evolutionary relationship of shattering genes in the studied cultivars based on phylogenetic analysis with NJ algorithm.

## Methods

### Identification of shattering genes

BRAD database (http://brassicadb.org/brad/) was used to retrieve protein, genomic, CDS and cDNA sequences of the 8 shattering genes *SHP1/2*, *FUL*, *ALC*, *NAC*, *IND*, *RPL*, and *PG* and their orthologs in *B.napus* and *B. juncea* [15]. Other databases like NCBI (https://www.ncbi.nlm.nih.gov/), TAIR (https://www.arabidopsis.org/), and Plants Ensembl (http://plants.ensembl.org/) were also consulted. A web tool from EMBL was used to identify different protein domains (http://smart.embl.de/smart/set_mode.cgi). The Basic local alignment search tool (BLAST) (htpp://www.ncbi.nlm.nih.gov/BLAST/) was used to search the homology of the shattering genes in *B.napus* and *B.juncea*. ProtParam tool was used to study the primary structure of shattering genes (http://expasy.org/tools/protparam.html). The gene structure display server (GSDS) web tool was used to align the CDS sequences of shattering genes with genomic sequences to identify exons and introns [16].

### Phylogenetic analysis of shattering proteins

*B. napus* and *B. juncea* shattering protein sequences were obtained from the BRAD database using reference sequences of shattering genes obtained from the TAIR database and the other plants protein sequences were obtained from the NCBI database and then aligned using the Clustal X program [17]. Using the Neighbor Joining (NJ) algorithm, a phylogenetic tree was constructed with MEGA 11 software [18]. The implication of nodes was calculated using a bootstrap study of 1000 replicates. For the surety of different domains that show the topology of NJ tree, pairwise gape deletion mode was used.

### Analysis of conserved motifs in shattering proteins

MEME software (Multiple Em for Motif Elicitation, V4.9.0) was used to analyze MADS-box shattering genes protein sequences as described by Bailey et al. [19]. MEME search was run with the following parameters: (1) maximum number of motif identification = 10; (2) optimum motif width > 6 and < 200.

### Chromosomal locations analysis

To find out identical genes, all shattering genes of *B. napus* and *B. juncea* were BLAST searched (htpp://www.ncbi.nlm.nih.gov/BLAST/) against each other, with a query coverage and similarity percentage of candidate genes of more than 80% [20]. The *Brassica* database (http:// brassicadb.org/brad/) was used to acquire positional information for all putative shattering genes along the 10 chromosomes of *B. napus* and *B. juncea* [15]. All genes were mapped along the 10 chromosomes, and the gene’s location was observed.

### Analysis of syntenic relationships

The comparative genomic synteny was performed to find the relationship among distinct shattering genes like *SHP1/2, FUL, ALC, NAC*, *IND*, *RPL*, and *PG* in *B.napus* and *B. juncea* using the circoletto program; genome visualization tool circoletto [14].

### Plant collection and sample preparation

Seeds of two *Brassica* varieties canola (Punjab Sarson) and Indian mustard (Super Raya) were collected from the plant Bioresources Conservation Institute (BCI) and Crop Sciences Institute (CSI) of the National Agricultural Research Centre (NARC), Islamabad Pakistan. The seeds were cultivated at National Institute for Genomics and Advanced Biotechnology (NIGAB), National Agriculture Research Centre (NARC), Islamabad, Pakistan under pods in a glasshouse. Forty days old samples of pre-mature and mature siliques of *B. napus* (Punjab Sarson) and *B. juncea* (Super Raya) were collected and stocked at – 80 °C for gene expression analysis. Morphological analysis was performed, and the data of plants were recorded in triplicates.

### RNA extraction and cDNA synthesis

Total RNA from the fresh and mature siliques of *B. napus* and *B. juncea* was extracted using a Pure Link^TM^ RNA Mini kit (Invitrogen). The RNAs were quantified by using BioSpec-nano Micro-volume UV-Vis Spectrophotometer (Shimadzu). The quality and integrity of RNA was checked on 1.5% agarose gel. cDNA was synthesized by using RevertAid^TM^ reverse transcriptase enzyme (Fermentas^TM^ Cat.No. K1621) following the manufacturer’s guidelines.

### Expression analysis of Shattering genes qRT-PCR

The expression pattern of shattering genes (*SHP1/2*, *FUL*, *ALC*, *NAC*, *RPL*, *PG*, and *IND*) was determined in fresh and mature silique of *B. napus* and *B. juncea* using comparative ΔCT method in real-time PCR (Applied Biosystems) with StepOnePlus software. For the execution of a relative expression, the Elongation factor (EF) was used as endogenous control. No template control (NTC) was also used as negative control in the assay. In total, 10 μl reaction volume, 5 μl Maxima SYBER Green (Thermo Fisher) genes specific primers (1 pmol of each), and 1 μl cDNA as a template were used. Real-time PCR conditions set were; denaturation at 94 °C for 10 min, the second stage followed by 40 cycles at 95 °C for 40 s, 58 °C for 32 s 72 °C for 32 seconds. Finally, a melt curve study was carried out at 52 °C to 95 °C. The statistical analysis of results was carried out by mean of relative fold expression of transcript ± standard deviation (SD). All the primers used in the qRT-PCR analysis listed in Table 1 were designed manually through the conserved region from the A and C subgenome of *B. napus*. The length of the amplified fragment ranged between 100 and 130 bp.

**Table 1:** Primers used for qRT-PCR analysis of shattering genes in *B. juncea* and *B. napus* fresh and mature siliques

Sr. no	Primer name	Primer sequences 5′–3′	Product size
1	SHP1-F	GTAGTCACGACGCAGAGAGTA	80
	SHP1-R	AACTTCAGCATCACACAAGAC	80
2	SHP2-F	GTGTAAGAGGAACGATCGAAA	81
	SHP2-R	TCACCAAGAATGTGTCTGTTC	81
3	FUL-F	GACTCTTGCATGGAAAGCATA	82
	FUL-R	TCTTCTCAAGTACCTCAACTC	82
4	IND-F	GAAACCCTAAGCCACTTCCAG	81
	IND-R	CTCGCTTATCCTTTCTCTAC	81
5	NAC-F	GGGCAGCAACTTCTGGTTACT	85
	NAC-R	TCAGTGAGGCGATATTCATGC	85
6	ALC-F	GTTTCCTCCGCTGAGATGTTC	81
	ALC-R	ATGAATTTCGCTGTCTAGCTC	81
7	RPL-F	GTGTGGGTCATGGTATTTACA	84
	RPL-R	ATACCTCTTGTAAACCTCGTC	84
8	PG-F	GTGTGGAAGTCTCTCCAAATC	84
	PG-R	ACACAGAGGGAGTAGCTTGCC	84
9	EF	CCAAGAATGGGCTTTATGC	130
		GTGATAGAGTGTCCAACAAGGTAAGTA	

## Results

### Identification and sequence analysis of shattering genes

A set of 32 individual shattering genes orthologues from *B. napus* and *B.* juncea genome was retrieved and their annotations were checked using keyword gene id to search Swissport annotations at the *Brassica* database (BRAD) (http://brassicadb.org/brad/). These genes were in greater number than those of model plant *Arabidopsis thaliana* as shown in Tables 2 and 3. The domain of these shattering genes was also identified using EMBL (http://smart.embl.de/smart/set_mode.cgi). The first six shattering genes of *B. napus* (Br-nS1-Br-nS6) contain the MADS-box domain whereas, 7–10 contain HLH, 11, 12, Pfam, 13, 14 pox/Hox 15–17 contain PbH1 domain. In *B. juncea*, 18–22 contain MADS-box domain while 23–26 HLH, 27, 28 Pfam, 29, 30 pox/Hox and 31, 32 PbH1 domain. We selected 32 annotated shattering genes of *B.* napus and *B. juncea* as Br-nS1 and Br-jS1 followed by Arabic numbers 1–32.In total shattering genes, 11 were mad box genes having mad box domain whereas the other 21 genes did not possess mad box domain. The genes that lacked mad box domain shared a large sequence resemblance with mad box protein of other crop varieties that also lack this domain and are considered to be mad box or shattering genes. Sequence analysis showed that all shattering genes of *B. napus* and *B. juncea* have introns except the gene *IND*. The maximum numbers of introns were identified in MADS-box shattering genes. These appearances are persistent with shattering genes previously determined in Arabidopsi*s* thaliana and *Brassica rapa.*

**Table 2:** In silico study of 17 shattering genes identified in *B. napus* with their closest *Arabidopsis* homologs and sequence feature

Gene name	Gene locus	Chr. no.	Closest Arabidopsis homologs	Protein length	Mol. wt. (kda)	PI	Introns
BrnS1	GSBRNA2T00098954001	A07	SHP1/AGL1	348aa	39.77	9.21	8
BrnS2	GSBRNA2T00105875001	C06	SHP1/AGL1	248aa	28.41	9.11	6
BrnS3	GSBRNA2T00132708001	A05	SHP2/AGL5	244aa	28.00	9.21	5
BrnS4	GSBRNA2T00113594001	A03	FUL/AGL8	241aa	27.43	9.37	7
BrnS5	GSBRNA2T00094717001	A09	FUL/AGL8	241aa	27.49	9.31	7
BrnS6	GSBRNA2T00086507001	C02	FUL/AGL8	241aa	27.45	9.36	7
BrnS7	GSBRNA2T00070429001	C07	ALC/AT5G67110/BHLH73	216aa	23.51	9.03	4
BrnS8	GSBRNA2T00063470001	C02	ALC/AT5G67110/BHLH73	98aa	11.12	10.0	5
BrnS9	GSBRNA2T00153545001	C03	IND/EDA33/GT10	178aa	20.36	7.93	1
BrnS10	GSBRNA2T00112126001	A03	IND/EDA33/GT10	182aa	20.60	6.06	0
BrnS11	GSBRNA2T00150558001	A10	NAC/At5g22380/MWD9.18	285aa	32.25	6.91	2
BrnS12	GSBRNA2T00085330001	C05	NAC/At5g22380/MWD9.18	286aa	32.37	7.60	2
BrnS13	GSBRNA2T00069510001	A10	BLH9/RPL/BLR/LSN/PNY	578aa	62.49	7.12	4
BrnS14	GSBRNA2T00088804001	C02	BLH9/RPL/BLR/LSN/PNY	575aa	61.96	6.94	4
BrnS15	GSBRNA2T00064043001	C08	PG/At1g45015	419aa	43.97	8.83	3
BrnS16	GSBRNA2T00052454001	A09	PG/At1g45015	418aa	43.88	8.83	3
BrnS17	GSBRNA2T00089606001	A08	PG/At1g45015	420aa	43.85	8.39	3

*aa* amino acid, *kda* kilo Dalton

**Table 3:** In silico study of 15 shattering genes identified in *B. juncea* with their closest *Arabidopsis* homologs and sequence feature

Gene name	Gene locus	Chr. no	Closest Arabidopsis homolog	Protein length	Mol. wt.(kda)	PI	Introns
BrjS18	BjuB022348	B06	SHP1/AGL1	278aa	31.74	8.49	6
BrjS19	BjuB022350	B06	SHP1/AGL1	247aa	28.20	9.11	6
BrjS20	BjuB001727	B01	SHP2/AGL5	244aa	28.00	9.12	5
BrjS21	BjuB027201	B04	FUL/AGL8	159aa	18.50	9.62	4
BrjS22	BjuB037752	B02	FUL/AGL8	301aa	34.82	9.08	7
BrjS23	BjuB020848	B06	ALC/AT5G67110/BHLH73	222aa	24.59	9.62	4
BrjS24	BjuA011758	A07	ALC/AT5G67110/BHLH73	214aa	23.41	9.37	4
BrjS25	BjuB019604	B08	IND/EDA33/GT10	191aa	21.62	5.97	0
BrjS26	BjuB019326	B08	IND/EDA33/GT10	191aa	21.59	5.97	0
BrjS27	BjuA038017	A10	NAC/At5g22380/MWD9.18	285aa	32.25	6.91	2
BrjS28	BjuB030790	B03	NAC/At5g22380/MWD9.18	293aa	32.96	6.46	2
BrjS29	BjuB001605	B08	BLH9/RPL/BLR/LSN/PNY	577aa	62.00	8.85	3
BrjS30	BjuA040195	A10	BLH9/RPL/BLR/LSN/PNY	586aa	63.15	6.95	3
BrjS31	BjuA029936	A08	PG/At1g45015	420aa	43.74	7.96	3
BrjS32	BjuB032977	B03	PG/At1g45015	421aa	43.74	7.93	2

*aa* amino acid, *kda* kilo Dalton

### Phylogenetic analysis of shattering genes

**Fig. 1:**
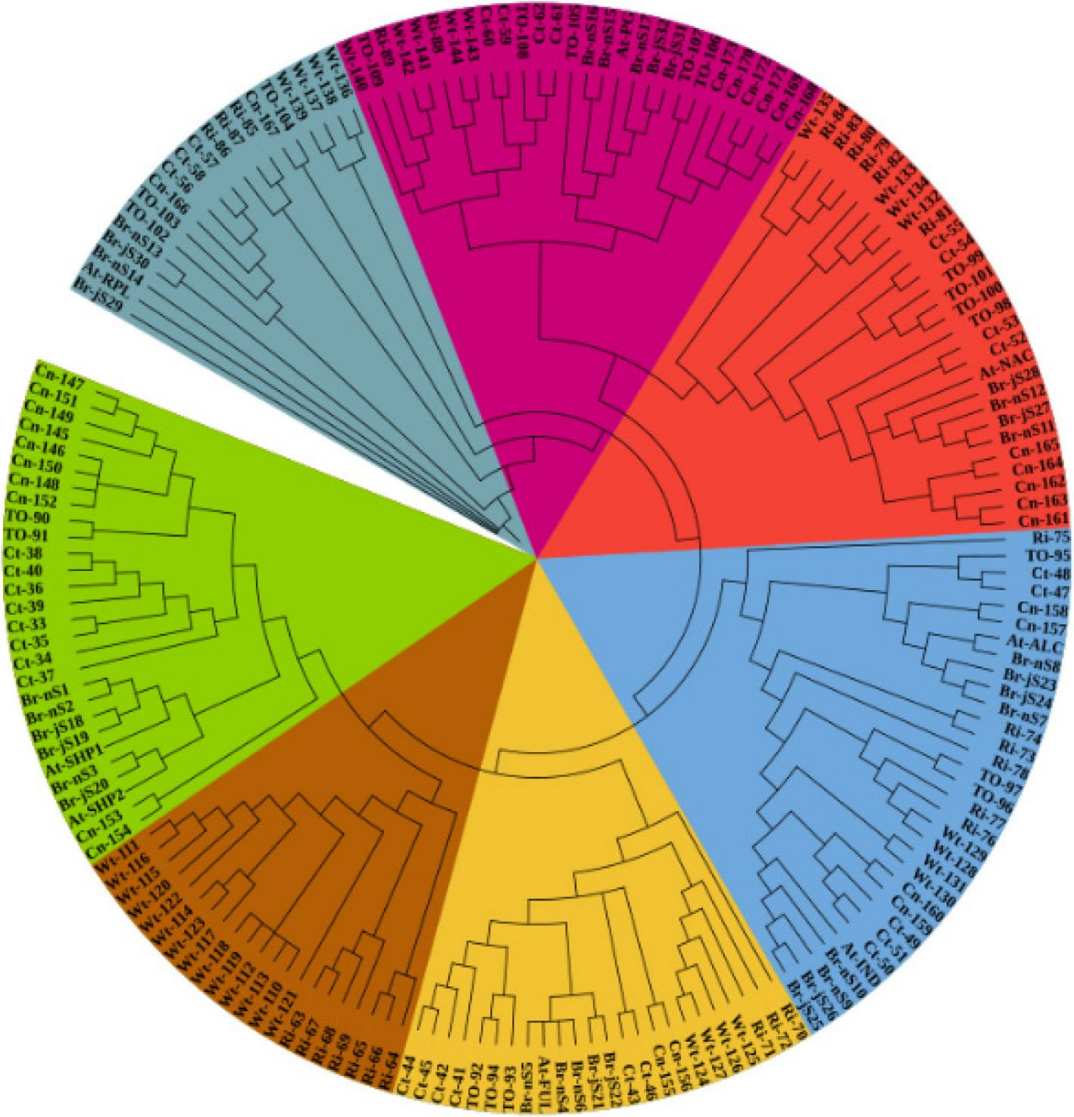
Neighbor joining consensus phylogenetic tree of 181 shattering genes from *B. napus* (17), *B. juncea* (15), Arabidopsis (At 8), citrus (Ct 30), cotton (Cn 29), rice (Ri 27), wheat (Wt 35), and tomato (TO 20)

The identified shattering genes protein sequences were used to analyze the phylogenetic relationship of the shattering gene family in *B. napus*, *B. juncea*, Arabidopsis, citrus, tomato, wheat, cotton, and rice. The unrooted phylogenetic tree characterizes the length of clades and the level of the evolutionary relationship with well-supported bootstrap values. The sequences of shattering genes *SHP1*, *SHP2*, *FUL*, *IND*, *ALC*, *NAC*, *RPL*, *PG* and their orthologous determined into *B. juncea*, *B. napus*, Arabidopsis, citrus, tomato, wheat, cotton and rice were aligned to generate the NJ phylogenetic tree (Fig. 1). Every individual shattering gene organized in a distinct clade with various color, characterize their functional and sequential conservation. The light green and dark brown colour in the tree is Clade I which contains a duplication of *SHP1* and *SHP2* in various plant like *B. napus*, *B. juncea*, citrus, tomato, wheat, cotton and rice except *SHP2* gene where no duplication was observed in *B. napus* and *B. juncea* plants. However, clade II with yellow color consists of *FUL* genes where duplications were observed. This shows that clades I and II are closely related to each other as compared to other clades. Clade III indicated with blue color, shows duplication and triplication of *IND* and *ALC* in *B. juncea*, *B. napus* and other plants like citrus, tomato, wheat, cotton and rice that indicates divergence in sequences and clade IV with red colour, duplication of *NAC* genes was observed. It is clear from the resulting tree that clade III and clade IV are closely related to clade I and II. Similarly, clade V with purple colour and clade IV with dark blue colour contains *PG* and *RPL* genes in a duplicated form in *B. napus*, *B. juncea*, and all other plants. The clade comprising *SHP1* and *SHP2* genes contains a greater number of genes as compared to others. Genes from these two clades are present on different chromosomes indicate that every individual gene bear duplication and whole genome triplication events before reaching this level. Environmental, physiological, and chromosomal rearrangement at the development level brought changes in the genome. These results authenticate that every individual gene of *B. napus*, *B. juncea*, citrus, tomato, wheat, cotton, and rice under observation are shattering genes having a close resemblance to each other and with a model plant *Arabidopsis thaliana* as shown in Fig. 1.

### Gene structure organization and conserved motifs analysis of shattering proteins

**Fig. 2:**
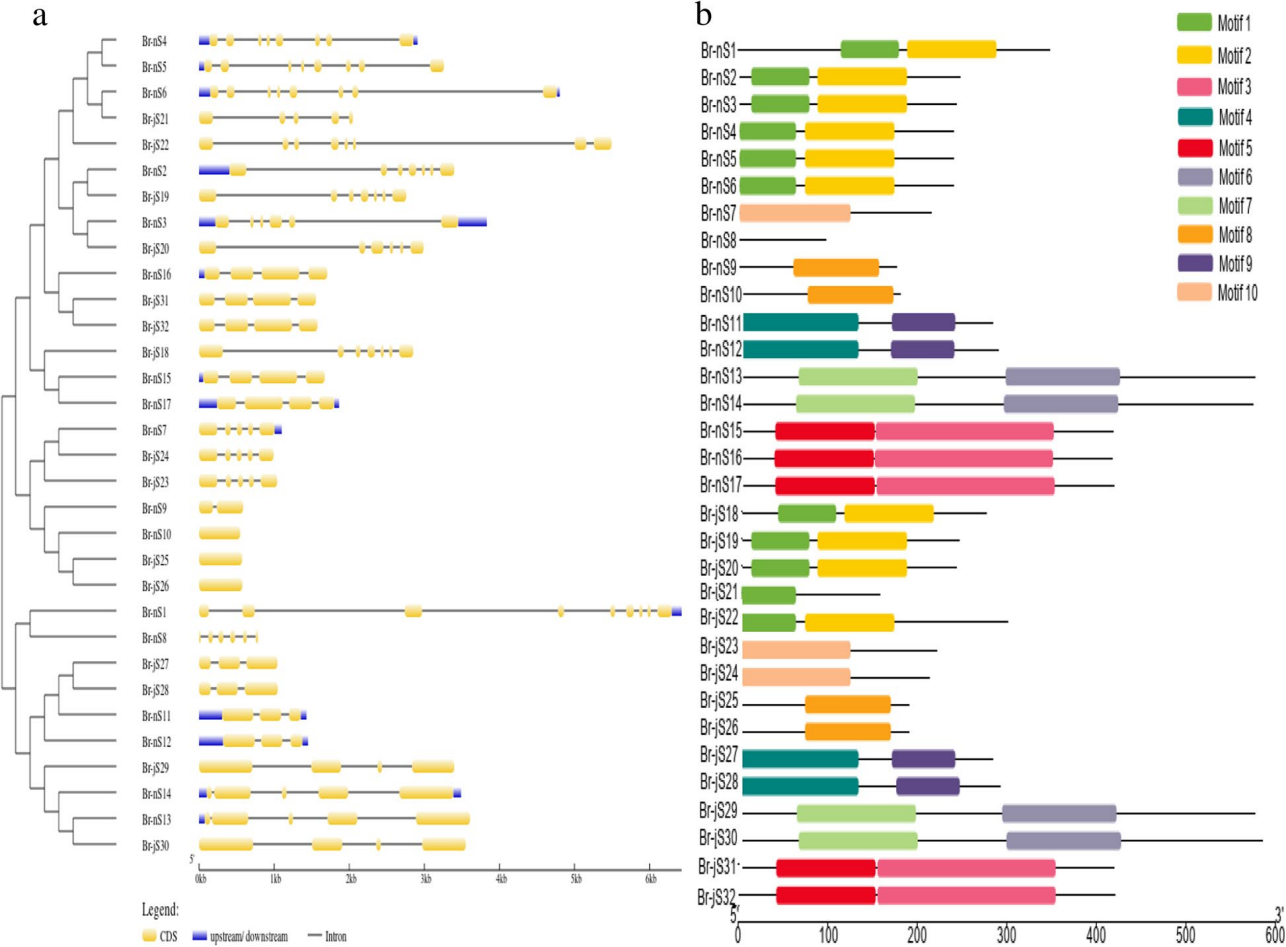
**a** Exon–intron structure of shattering genes in *B. napus* and *B. juncea*. Exons are shown as yellow boxes, introns are shown as a thin black line, and UTRs are shown as blue boxes. **b** Motif distribution analysis, 10 motifs are shown as colored boxes

We compared the coding DNA sequences of exons and introns to their genomic DNA sequences to facilitate phylogenetic reconstruction. As shown in Fig. 2a, the distribution, number, and length of exons and introns were not highly diverse among all genes. Br-nS1 was the longest sequence and Br-nS10 was the shortest among all shattering genes. Br-nS1 contain 8 introns. While Br-nS2, Br-jS18, and Br-jS19 contain six introns. Br-nS4, Br-nS5, and Br-nS6 contain seven introns while in *B. juncea* divergence is found Br-jS21 contains 4 and Br-jS22 contains 7 introns for the same gene. Br-nS9 contain a single intron, whereas the same gene Br-nS10, Br-jS25 and Br-jS26 didn’t contain any introns and showed a divergence in the gene sequence. Br-nS13 and Br-nS14 contain four introns whereas the same genes in *B. juncea*, Br-jS29, and Br-jS30 contain three introns showing a clear difference among *B. napus* and *B. juncea*. Similarly, Br-nS15, Br-nS16, and Br-nS17 genes contain three introns, while in other species *B. juncea* Br-jS31 contain three and Br-jS32 contains two introns for the same genes, which also showed some variance in genes sequences.

MEME (Multiple Em for Motif Elicitation) motif search tool was used to identify 10 conserved motifs of 32 shattering protein sequences of *B. napus* and *B. juncea* (Fig. 2b). Motifs 1 and 2 exhibit the MADS-box domain which was found in 11 genes whereas other shattering genes did not show motif 1 or 2 features. The genes which exhibit the characteristics of motifs 1 or 2 were Br-nS1-Br-nS6 and Br-jS18-Br-jS22. These genes did not contain other representative motifs of Mads-box family such as motifs 4, 5, 6, 7, 8, 9, and 10. Motif 4 and 5 comprised of PbH1 domain found in 5 genes which were Br-nS15, Br-nS16, Br-nS17, Br-jS31, and Br-jS32. Br-nS7, Br-nS9, Br-nS10, Br-jS21, Br-jS23 Br-jS24, Br-jS25, and Br-jS26 genes consists of single motif whereas Br-nS8 gene did not contain any motif. Motif 8 and 10 showed pox/Hox domain which was found in Br-nS13, Br-nS14, Br-jS29, and Br-jS30 gene. Br-nS15, Br-nS16, Br-nS17, Br-jS31, and Br-jS32 comprised PbH1 domain with motif 5 and 6 features. Motif 1 and Motif 2 were conserved among genes which is the characteristic feature of shattering genes. The different motifs are represented by different colors that showed similarities among *B. napus* and *B. juncea* as shown in (Fig. 2b). The number of motifs found in both species is similar except for Br-nS7, Br-nS9, Br-nS10, Br-jS21, Br-jS23 Br-jS24, Br-jS25, and Br-jS26 which shows single motif and revealed similarities and differences with other shattering genes among *brassica* species.

### Chromosomal distributions of shattering genes

According to the available *Brassica* genome database, 32 shattering genes were mapped onto 10 chromosomes of *B. napus* and *B. juncea*. The chromosome localization of *Brassica* gene ID was confirmed by ensemble Plant Browser. There were 17 shattering genes distributed on both the A and C subgenome of *B. napus* while on the B and A sub-genome of *B. juncea*, 15 shattering genes were observed. Similarly, on the A subgenome of both plants, a total of 13 genes were identified and on the B sub-genome of *B. juncea*, 11 genes were observed, while on the C genome of *B. napus*, 8 genes were observed.

**Fig. 3:**
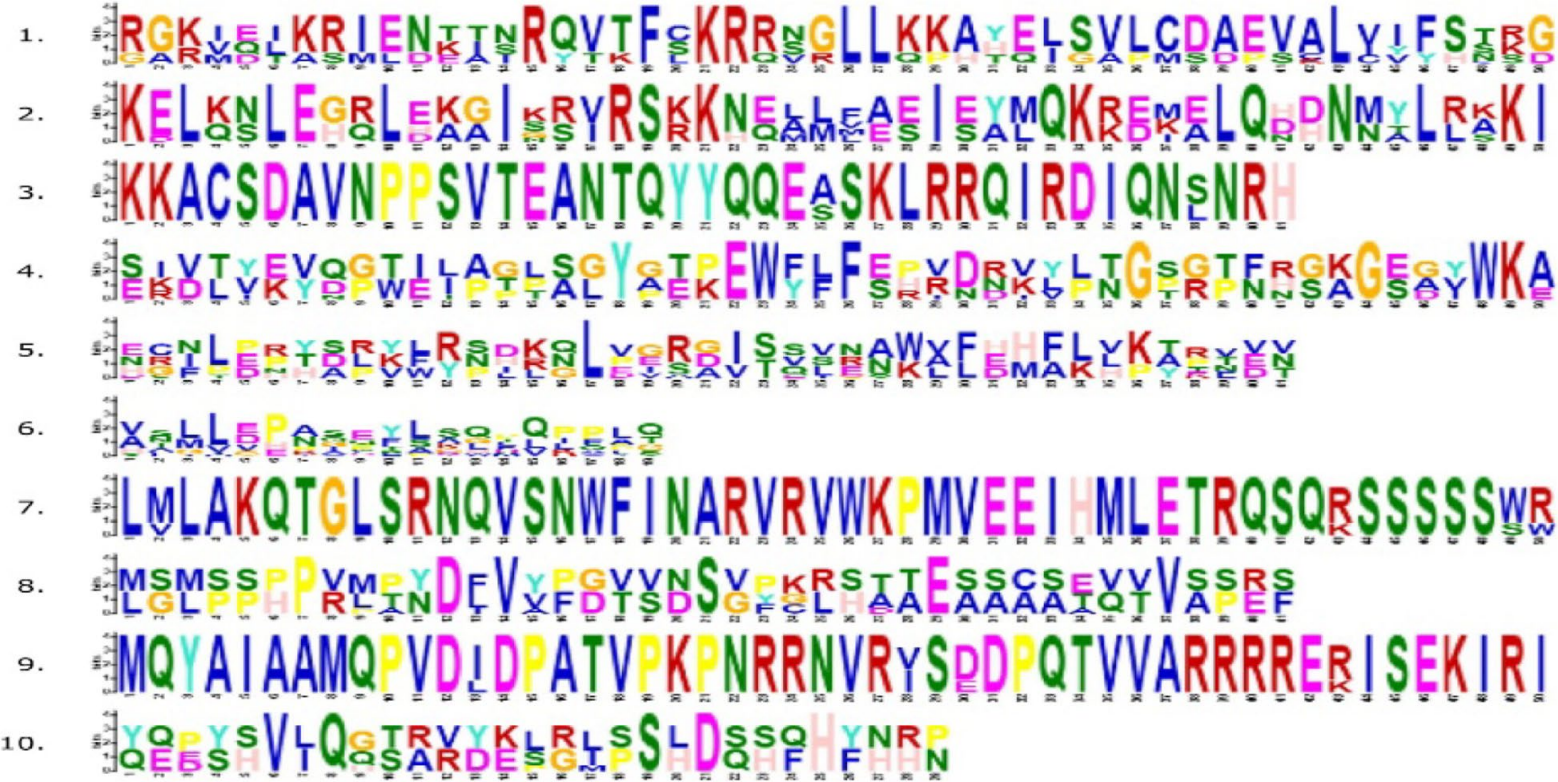
Logos of tens motifs discovered in shattering genes

**Fig. 4:**
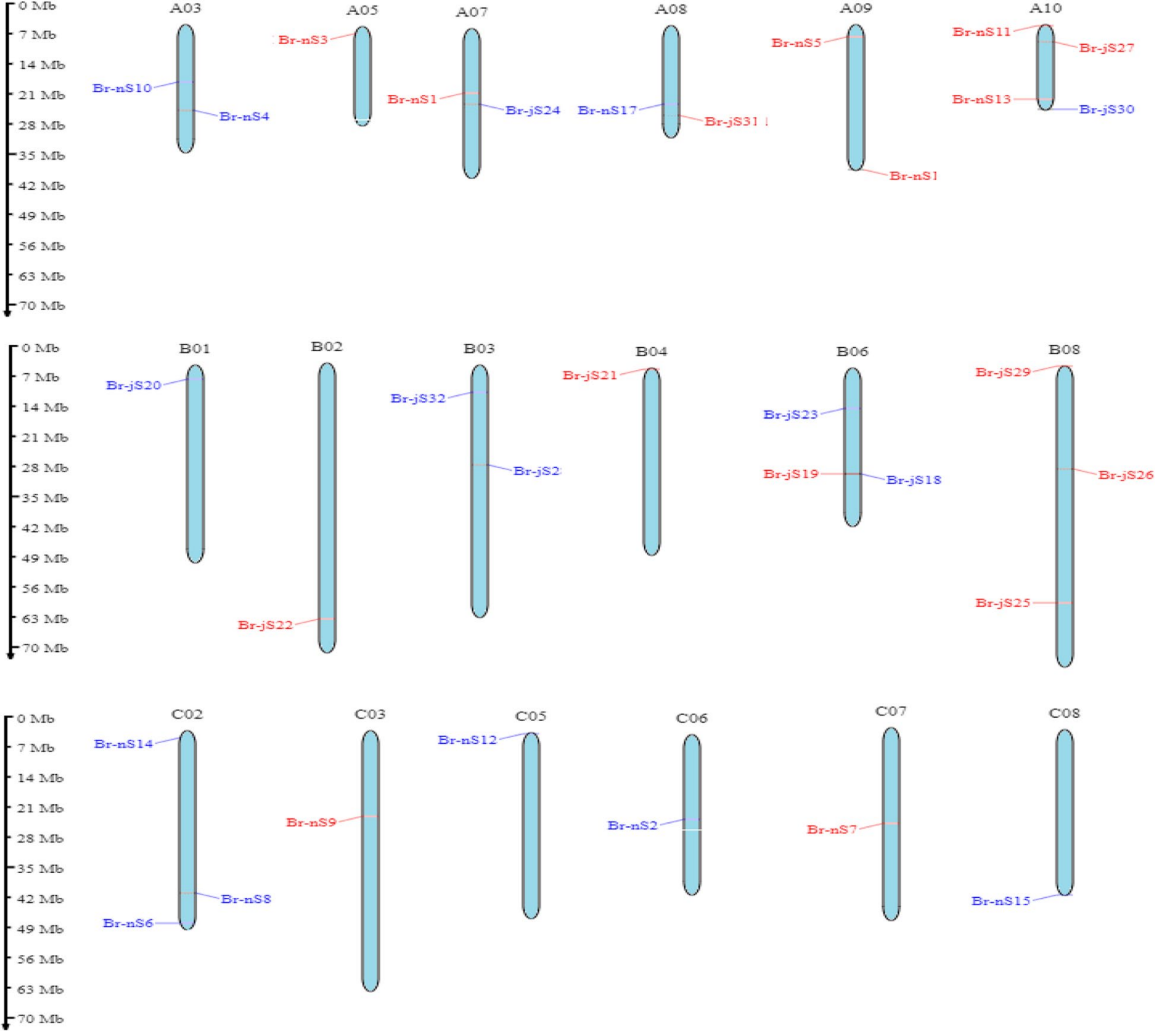
Gene localization of shattering genes on *B. napus* and *B. juncea* chromosomes

According to our results, Br-nS4 and Br-nS10 genes were observed on similar chromosome A03, whereas Br-nS3 lies on chromosome A05. Similarly, Br-nS1 and Br-jS24 were identified on the same chromosome A07, while Br-nS17 and Br-jS31 were located on chromosome A08. Genes like Br-nS5 and Br-nS16 were located on the A09 chromosome, whereas Br-nS11, Br-nS13, Br-jS27, and Br-jS30 were observed on chromosome A10. Similarly, on chromosome B01, gene Br-jS20 was observed while on the B02 chromosome, gene Br-jS22 was located. Br-jS28 and Br-jS32 genes were identified on the same chromosome B03, whereas on B04 chromosome Br-jS21 gene was located. Hence genes Br-jS18, Br-jS19, and Br-jS23 were observed on similar chromosome B06. Similarly, on the B08 chromosome, genes like Br-jS25, Br-jS26, and Br-jS29 were identified. The genes observed on chromosome C02 were Br-nS6, Br-nS8 and Br-nS14, whereas on other chromosomes like C03, C05, C06, C07, and C08, genes located were Br-nS9, Br-nS12, Br-nS2, Br-nS7, and Br-nS15 respectively. Hence, all the shattering genes were scattered on *Brassica* chromosomes as shown in Figs. 3 and 4.

### Syntenic relationship among shattering genes of B. napus and B. juncea

**Fig. 5:**
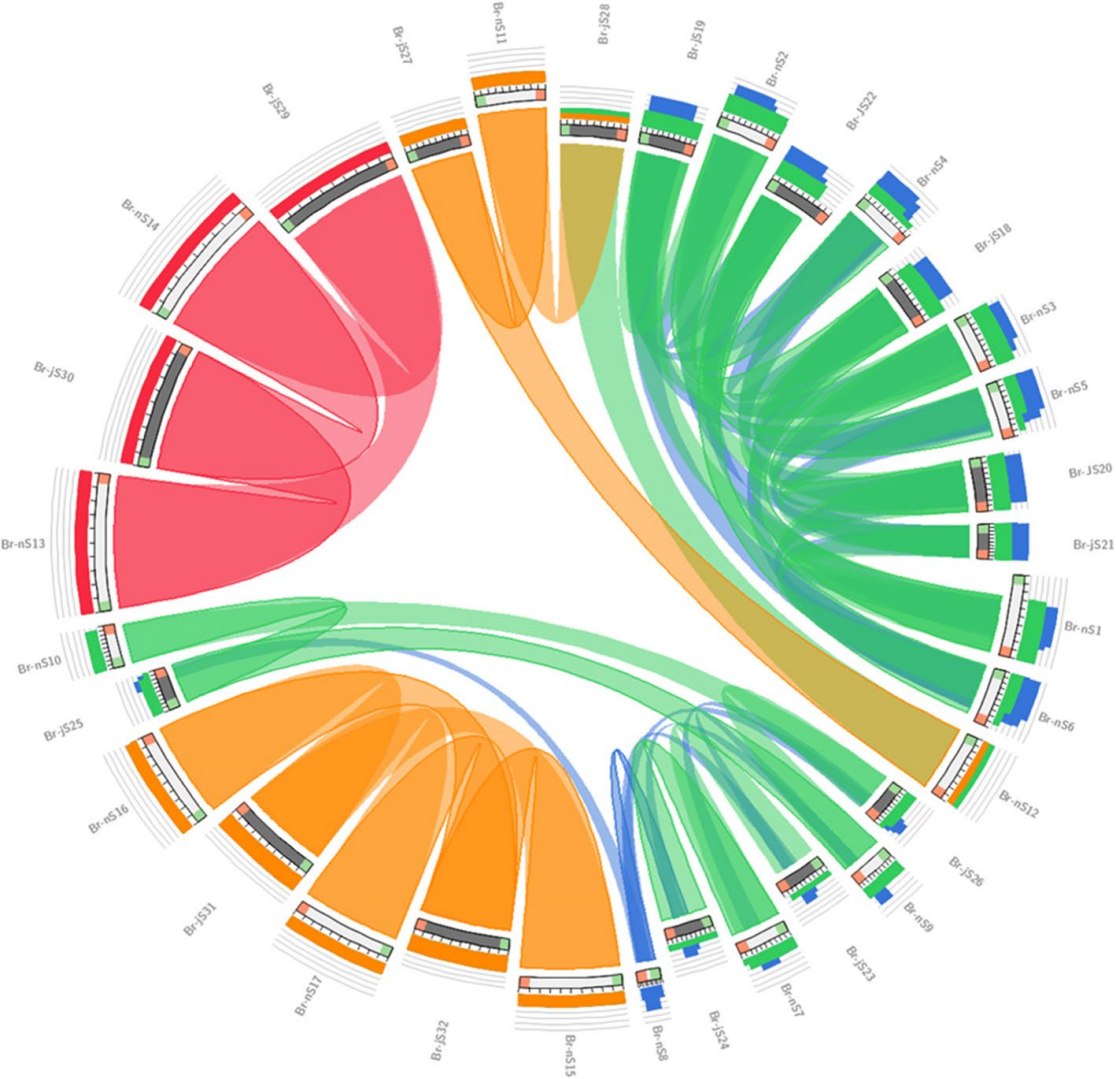
Representation of synteny of *B. napus* and *B. juncea* identifying the level of conservation at the sequence level in 4 colors. The red, green, orange, and blue colors signify the level and intensity of evolutionary conservation among distinct shattering genes, e.g. maximum intensity is from orange to green

Comparative genomic synteny analysis was performed by circoletto tool (tools.bat.inspire.org/circoletto/) for genome conservation visualization. The orthologs’ relationship and conservation were determined for the shattering gene family in *B. napus* and *B. juncea.* Synteny diagram represents a remarkable relationship among these species in the context of duplication, triplication, evolution, function, and expression (Fig. 5) showed a unique relationship among *B. juncea* and *B. napus.* It was observed that *B. napus* Br-nS13 and Br-nS14 gene sequence showed synteny with *B. juncea* sequence Br-jS29 and Br-jS30, while *B. napus* gene sequence Br-nS15, 16, and 17 showed synteny with *B. juncea* gene sequence Br-jS31, 32 and gene sequence Br-nS11 and 12 showed synteny with Br-jS27 and Br-jS28. In Addition, Br-nS7 and Br-nS8 gene sequence showed synteny with Br-jS23 and Br-jS24 gene sequences while Br-nS9 and Br-nS10 showed synteny with Br-jS25 and Br-jS26 gene sequences. Similarly, Br-nS1 and Br-nS2 showed synteny with Br-jS18 and Br-jS19 gene sequences, while Br-nS3 showed synteny with Br-jS20. *B. napus* gene Br-nS4, 5, 6 sequences showed synteny with Br-jS21 and Br-jS22. In comparative synteny analysis inward tangling ribbons color intensity exhibited the rate of conservation while outward tangling ribbons showed duplication events. Genomic dynamicity and evolutionary improvement along mobile elements in the genome of *B. napus* and *B. juncea* were determined in syntenic circles. In chromosomal shuffling, duplication, and triplication mobile elements play an important role. A permanent position was adopted by the blocks at a specific position in genome initiate expression that involve another biological pathway disturbance (Fig. 5).

### qRT-PCR expression of shattering genes in fresh and mature siliques

**Fig. 6:**
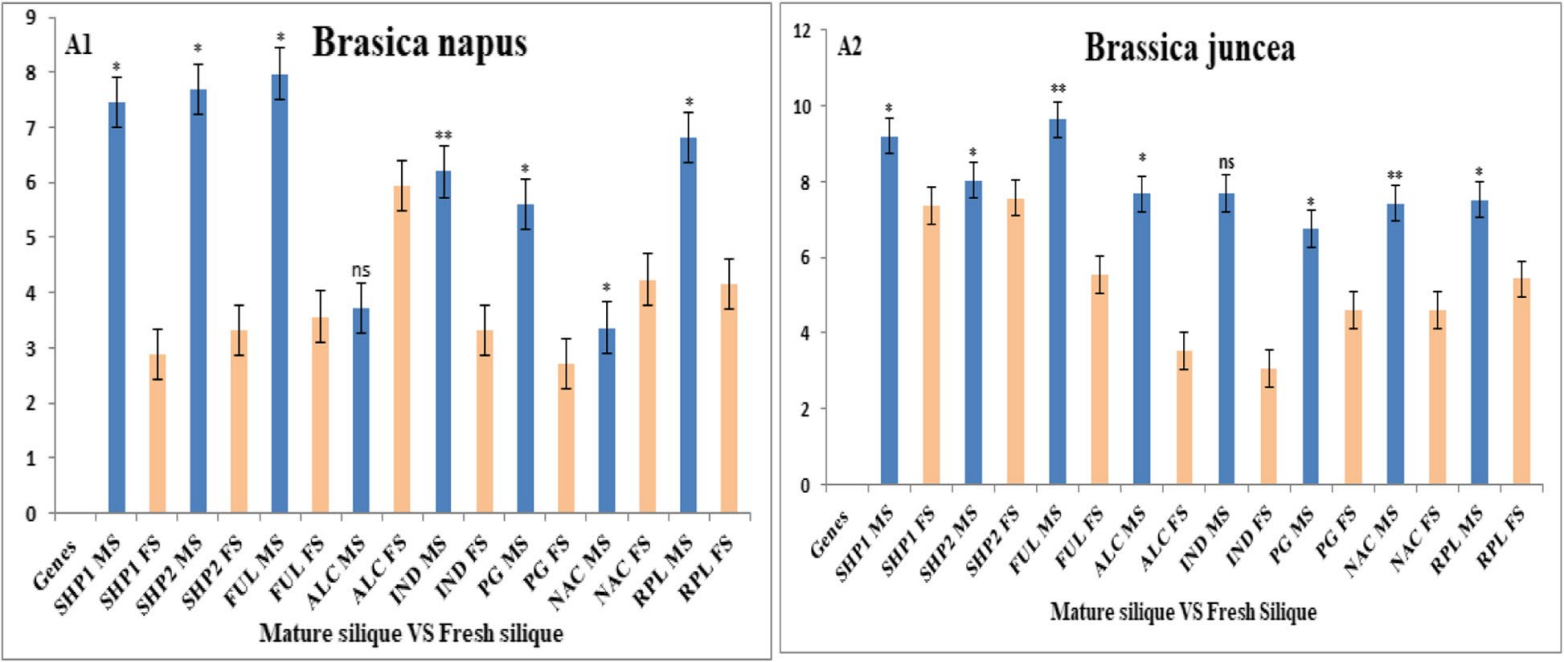
The expression levels of shattering genes in *B. napus* and *B. juncea*: Graph bar defining the difference and the correlation in the expression level among two tissues of *B. napus* and *B. juncea*. **A1** A lower level of shattering genes expression in *B. napus* than *B. juncea*. **A2** A higher level of shattering genes expression in *B. juncea* than *B. napus* in the given tissues. *ALC* gene expressed more in *B. napus* fresh silique. Lower expression of *ALC* gene was observed in fresh silique of *B. juncea* but highly expression was observed in mature silique

The expression levels of shattering genes in fresh and mature siliques of *B. napus* and *B. juncea* were confirmed by qRT-PCR. Our results inferred that the expression level of shattering genes was higher in *B. juncea* as compared to *B. napus* in both fresh and mature siliques. Strong signals of shattering genes were observed in mature siliques in both species, while in fresh silique, the transcripts levels were low (Fig. 6). The correlation is completely noticeable in the evidence that shattering genes play a major role in shattering associated pathways by devoting to developmental pathways of lignification and valve margin associated transcriptional activity. Moreover, *ALC* gene expression was upregulated in fresh silique of *B. napus* while down regulation of *ALC* gene was observed in fresh silique of *B. juncea*. Similarly, higher expression of *ALC* genes was observed in mature silique of *B. juncea* compared to *B. napus*. *PG* gene downregulation was observed in fresh silique of *B. napus* while it expressed more in *B. juncea*. The expression of *SHP1, SHP2*, *FUL* and *RPL* were observed more in *B. juncea* in both fresh and mature siliques showed a difference expression patterns.

## Discussion

*Brassicaceae* is a large plant family consists of ~ 338 genera and 3700 species, important both economically and agriculturally [19]. In addition to this, plants of this family are grown like a weed in different parts of the world including North America, South America, and Austria [21]. Arabidopsis thaliana, a model plant from the family *Brassicaceae* was the first plant to be entirely sequenced [21]. Plants and vegetables from this family offer essential food nutrients to human and other animals. Due to their great importance, all the *Brassica* plants have common and commercial value with a positive influence on earth and manhood. *Brassica* species have inconstant traits and morphological differentiation revealing that the genome of this family is very vibrant and endured a lot of rearrangement and evolutionary measures [22].

In this study, *SHP1*, *SHP2*, *FUL*, *IND*, *ALC*, *NAC*, *RPL*, and *PG* when compared at the genomic level showed close similarity. Protein and nucleotide shows an important correlation at the sequence level. It has been showed that these genes are responsible in shattering and seed development of plants [23]. The phylogenetic analysis here showed that *SHP1* as compared to other genes have fever dynamicity which is balanced in the connection of genomics but bear duplication. The duplicated genes determined with a distinct chromosome number in *B. napus* and *B. juncea* which recommended genomic flexibility as previously reported in Arabidopsis and *B. rapa* [24, 25] shows similar results with our investigations. *SHP2* shattering gene study uses a novel approach to phylogenetic analysis bears no duplication and triplication as previously reported in other *Brassica* species [26, 27]. *FUL* is known for fruit development in different *Brassica* species. The Phylogenomics of *FUL* affords unusually different results than *SHP1* and *SHP2*. The behavior observed more dynamics among the various species of *Brassica* family. *FUL* genes showing duplication and differential location in the genome of *B. juncea* and *B. napus* also previously described in *B. rapa* further strengthen our results [8].

In current research, we have study 32 shattering gene of *B. juncea* and *B. napus* which are more in number than the shattering genes reported for *A. thaliana* [28]. The syntenic analysis performed among *B. napus* and *B. juncea* shows the similar sequence feature and whole genome of both species go through triplication events since its divergence from Arabidopsis. The evolutionary and syntenic relationships among Arabidopsis and *B. rapa* is also supporting our results [29]. On the other hand, we observed the expression of shattering genes *SHP1*, *SHP2*, *FUL*, *IND*, *ALC*, *NAC*, *RPL*, and *PG* in *B. napus* and *B. juncea* like previously reported in Arabidopsis [30]. Our result also suggest that these genes are the reputed orthologous of Arabidopsis genes AGL1, AGL5, AGL8, AT5G67110, EDA33, At5g22380, BLH9, and At1g45015 might play the similar role, and they are expressed in both plants in fresh and mature siliques.

In previous studies, divergence in expression pattern was observed in shattering genes in *B. napus*. Wu et al. [31] determined the expression patterns and evolution of MADS-box TF family in *B. napus*. Becker and Theissen [32] reported that Shatterproof1/2 and genes which are members of MADS box family are engaged in controlling this pod shattering issue. *SHP1* and *SHP2* genes are involved in opening of silique in *B. napus* plants when the expression level is low [23, 33, 34].

The expression of these genes started from developed flower to mature silique with lower expression in the late stage of development of seed [31]. *SHP1*, *SHP2*, and *FUL* showed a relationship with *IND*, *ALC* that initiate acting to abrogate activity of DZ to forbid dehiscence at the time of seed formation follow indehiscence in the existence of multiple regulatory genes. The present analysis of all shattering genes showed different expression pattern in different tissues such as fresh and mature siliques of both plants as previously reported in Arabidopsis and *B. rapa*. These genes were expressed in both plant tissues, although in *B. juncea* they were slightly higher than in *B. napus*. These different expressions of shattering genes shows that they are important for cellular valve and margin evolution [24, 35].

A similar study was conducted by Yasin et al. [36], Ahmad et al. [37], & Khan et al. [38] whose results agree with our results. They demonstrated higher expression of *FUL* gene in mature aerial part silique plant as compared to leaves and flowers of *B. napus* plants. Similarly, *SHP1* and *SHP2* transcripts were expressed in flower silique whereas; no expression was detected in the leaves. Our findings showed basic gene expression information about shattering cascade genes which can be useful for developing genome edited *brassica* plants which are resistant to shattering.

## Conclusion

Conclusively, different orthologous of shattering genes are exists in the local cultivars of *Brassica*. After comparative phylogenetic study, molecular gene characteristics, motifs/domain identifications, and comparative expression study, it is validated that the sequences were conserved across *B. napus*, *B. juncea* as well as in *Arabidopsis* plant. The redundant expression was observed in fresh and mature siliques of both cultivars. The different expression patterns of shattering genes are also helpful to study the nature of both plants and their pathways related to transcription and regulation. Further analysis of shattering genes is required to uncover their functions involved in the regulation of different pathways.

## Data Availability

Not applicable.

## Abbreviations

μl: Microliter
μM: Miro molar
A01: Chromosome number for Brassica A genome
At: Arabidopsis thaliana
BJ: Brassica juncea
BLAST: Basic Local Alignment Search Tool
Bn: Brassica napus
Bp: Base pairs
C01: Chromosome number of Brassica C genome
cDNA: Complementary deoxyribonucleic acid
cm: Centimetre
Ct: Cycle threshold
NARC: National Agricultural Research Centre
NCBI: National Centre for Biotechnology Information
MEGA: Molecular Evolutionary Genetic Analysis
GL: Glucosinolates
