# Correction: Genome-wide identification, phylogeny, and expression profiling analysis of shattering genes in rapeseed and mustard plants

**DOI:** 10.1186/s43141-022-00419-z

**Published:** 2022-09-26

**Authors:** Mahideen Afridi, Khurshid Ahmad, Shahana Seher Malik, Nazia Rehman, Muhammad Yasin, Shujaul Mulk Khan, Adil Hussain, Muhammad Ramzan Khan

**Affiliations:** 1 National Centre for Bioinformatics, Quaid-I-Azam University, Islamabad, 45320 Pakistan; 2 Department of Biological Sciences, International Islamic University, Islamabad, 44000 Pakistan; 3 Department of Biology, College of Science, United Arab Emirates University, 15551 Al Ain, United Arab Emirates; 4 National Institute for Genomics and Advanced Biotechnology, National Agricultural Research Center, Park Road, Islamabad, 44000 Pakistan; 5 Department of Plant Sciences, Quaid-I-Azam University, Islamabad, 45320 Pakistan; 6 Food and Biotechnology Research Centre (FBRC), Pakistan Council of Scientific and Industrial Research (PCSIR) Laboratories Complex, Ferozepur Road, Lahore, Punjab 56400 Pakistan

**Correction: J Genet Eng Biotechnol 20, 124 (2022)**

**https://doi.org/10.1186/s43141-022-00408-2**

Following publication of the original article [1], the authors identified an error in the affiliation of Muhammad Yasin which is affiliated with affiliation 4 not 1. The correct affiliation is given in this article.

The author affiliation has been updated above and the original article [1] has been corrected.
